# Effects of phosphorus supply levels on the growth and nutrient utilization of *Pinus massoniana* lamb hybrid saplings

**DOI:** 10.3389/fpls.2025.1606643

**Published:** 2025-08-05

**Authors:** Wenyue Wang, Jinguo Hua, Zhen Zhang, Zhichun Zhou, Renchao Wu, Simin Qu, Xiuli Chu

**Affiliations:** ^1^ Research Institute of Subtropical Forestry, Chinese Academy of Forestry, Hangzhou, Zhejiang, China; ^2^ Zhejiang Key Laboratory of Forest Genetics and Breeding, Hangzhou, Zhejiang, China; ^3^ College of the Environment and Ecology, Xiamen University, Xiamen, Fujian, China; ^4^ Qingyuan Experimental Forest Farm, Qingyuan, Zhejiang, China

**Keywords:** *Pinus massoniana*, hybrid varieties, phosphorus fertilizer, interaction, shoot traits, nutrient utilization

## Abstract

**Introduction:**

Phosphorus is vital for plant growth but is often limited in subtropical acidic soils, restricting forest productivity. *Pinus massoniana*, a key timber and resin species in China, shows varied shoot-sprouting characteristics and nutrient use under different phosphorus levels. Understanding its response to fertilization is crucial for optimizing plantation management and growth.

**Methods:**

We employed a two-factor randomized block design with three *Pinus massoniana* hybrid varieties (with shared male or female parents) and four phosphate fertilizer levels (0, 100, 250, 500 g·plant⁻¹). After three growing seasons, tree height, diameter at breast height (DBH), annual shoot growth characteristics (fixed growth, free growth, shoot number), and growth amount were measured. Nitrogen and phosphorus contents in coniferous leaves were also analyzed.

**Results:**

Results showed significant differences in tree height, DBH, and fixed growth among varieties and fertilizer treatments. The 500 g·plant⁻¹ treatment performed best, with fixed growth 14.35% higher than the control. Free growth and shoot flushing frequency showed no significant differences among treatments. The interaction between variety and fertilization contributed 74.10% to free growth variation. Phosphorus fertilization enhanced growth and nutrient uptake in young *Pinus massoniana* saplings. Increasing fertilizer intensity raised nitrogen and phosphorus contents and the nitrogen-phosphorus ratio. Correlation analysis revealed that the nutrient environment influences genetic expression among traits, affecting their interrelationships.

**Discussion:**

This study examines how genotype, phosphate fertilizer intensity, and their interaction influence the growth and shoot-sprouting behavior of *Pinus massoniana* hybrids. Phosphate fertilization is a key driver of early growth and nutrient accumulation, while shoot-sprouting is largely shaped by variety-fertilizer interactions. Findings highlight the need to focus less on the genetic basis of shoot growth and more on how nutrient availability influences variety performance, supporting precise fertilization strategies for optimized forest management.

## Graphical Abstract

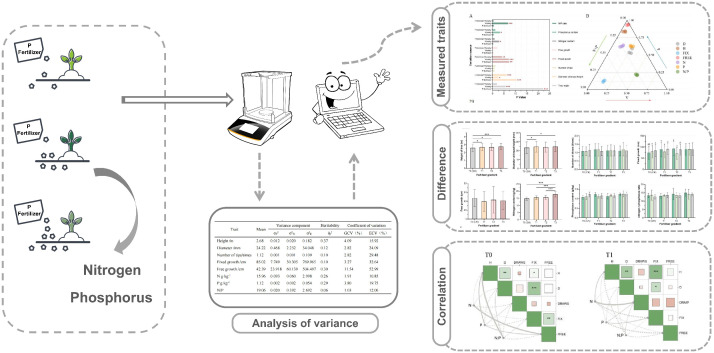

## Highlights

- Phosphorus fertilizer application significantly promoted the growth of *Pinus massoniana* hybrid varieties, increasing tree height by 6.14%, DBH by 5.49%, and fixed growth by 14.35% at the 500 g·plant^-^¹ application rate.
- The interaction between phosphorus fertilizer and variety significantly affected free growth, with a genetic coefficient of variation of 11.54% and an environmental coefficient of variation of 52.99%.
- Applying phosphate fertilizer is the key factor influencing the initial growth and nutrient accumulation of *Pinus massoniana* hybrids. Multiple shoot-sprouting behavior is primarily driven by the interaction between variety and phosphate fertilizer, while the nutritional environment affects genetic trait correlations.

## 1 Introduction

*Pinus massoniana* is one of the most important fast-growing timber species in subtropical China and a major resin-producing species, widely distributed in low hills and acidic red soil areas (Benarchid et al., 2022). However, these areas generally suffer from soil infertility and low phosphorus (P) availability, limiting the growth potential of major afforestation species such as *P. massoniana*, *Cunninghamia*, and *Eucalyptus* in the same region (Qiang et al., 2021). With the improvement of plantation management systems and the increase in intensive management levels, less land is needed to provide higher-yielding forest products (Klevtsov et al., 2021). Phosphorus is one of the essential elements for plant growth, playing a crucial role in cell energy metabolism, nucleic acid synthesis, root development and shoot architecture (Hanlon et al., 2018). In plantation management, the application of phosphorus fertilizer is usually an effective way to improve soil nutrients and promote forest growth (Sellin et al., 2024; Talboys et al., 2014). Currently, high-gain varieties obtained through breeding are mainly used in production, and fertilization during the sapling stage is the most commonly used plantation management measure (Bera et al., 2018). In the future, intensive or drone fertilization may become an important direction for plantation management (Gonzalez et al., 2013). Therefore, how to improve the growth potential of plantations through scientific fertilization and mastering the interaction between varieties and soil nutrients has become an important issue in current forestry research.

Shoot growth characteristics are a crucial component of tree growth patterns, directly influencing growth rate and wood productivity (Zhao et al., 2019; Zhang et al., 2020). Variations in shoot-sprouting behavior are a common strategy for *P. massoniana a* sapling to respond to changes in nutrient environments, depending on the effects of environment or genotype, or the interaction between genotype and environment (Martens et al., 2019). *P. massoniana* has entered the third round of genetic improvement, and genetic differences often lead to significant variations in tree height and diameter at breast height (DBH) among different varieties (Zhang et al., 2020). Although the genetic background of annual shoot traits may be somewhat offset by multiple rounds of genetic selection, the number of annual shoots and shoot growth characteristics are more susceptible to environmental factors (Zhao et al., 2019). For example, Martens et al. (2019) pointed out that pine trees exhibit a periodic growth pattern during annual growth, and free shoot growth is greatly affected by changes in environmental conditions. Especially when the nutrient environment is unstable, the free growth of trees will show greater environmental adaptability. Sellin et al. (2024) indicated that free shoot growth in trees is significantly affected by environmental conditions (such as nutrient environment), especially when the fertilization intensity is high, showing strong phenotypic plasticity, which is closely related to changes in different fertilization treatments. Different strains show significant differences in growth response to phosphorus fertilizer application, which is closely related to the role of genotype in nutrient absorption, utilization, and distribution (Guo W. et al., 2023). Specifically, phosphorus availability can alter the balance between primary and secondary shoot growth, as trees allocate resources to optimize nutrient capture under varying soil conditions. However, there are few studies on the interaction between high-generation hybrid varieties of *P. massoniana* and phosphorus fertilizer application on shoot traits, and the effects of genotype, phosphorus fertilizer, and their interaction on shoot-sprouting behavior and nutrient distribution have not been fully confirmed.

Needle nutrient content is widely recognized as a crucial indicator of tree adaptation to nutrient-poor environments, directly affecting tree growth rate, leaf longevity, and other processes, which are key to long-term tree productivity (Zhang et al., 2020). The nitrogen-to-phosphorus ratio (N/P ratio) is considered an important ecological indicator of plant nutrient limitation, closely related to forest growth strategies and adaptability (Guo Q. et al., 2023). The sensitivity of shoot-sprouting behavior and mechanisms to nutrient environments varies among genotypes, and the correlation between changes in shoot-sprouting behavior and needle nutrient content has not been thoroughly investigated. Therefore, we selected three hybrid varieties and established four phosphorus fertilizer application levels to quantify the growth characteristics, annual shoot behavior, and needle nutrient dynamics of *P. massoniana* saplings under different phosphorus fertilizer intensities. The main objectives were: (1) to investigate the effects of genotype, phosphorus fertilizer intensity, and their interaction on the growth and shoot growth characteristics of *P. massoniana* saplings; (2) to reveal the regulatory mechanism of phosphorus fertilizer on needle nutrient accumulation and utilization patterns; and (3) to establish the relationship between phosphorus fertilizer levels and genotype adaptability, providing a basis for *P. massoniana* variety promotion and precision fertilization management.

## 2 Materials and methods

### 2.1 Study area

**Figure 1:**
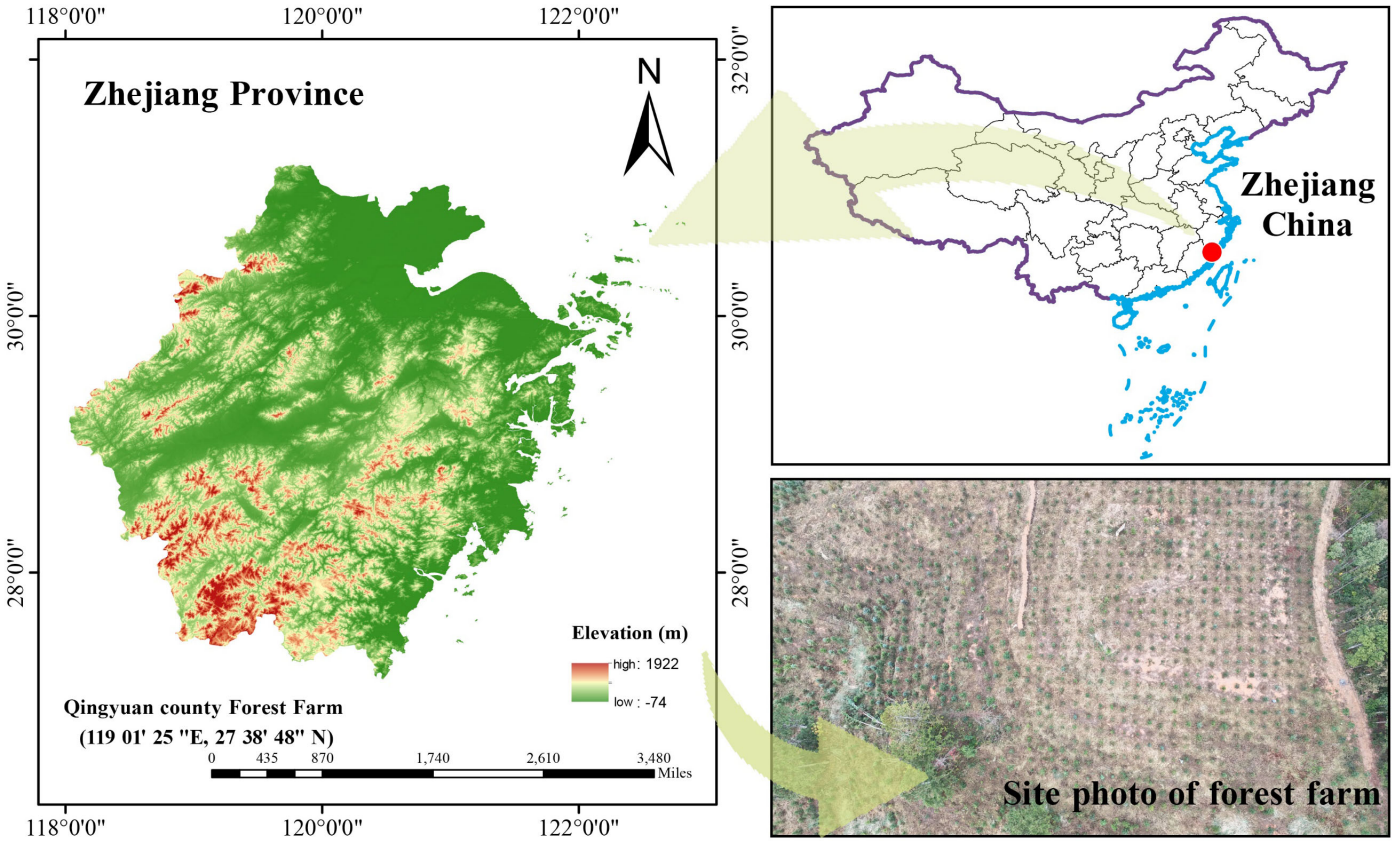
General situation of study area.

The experiment was conducted at the Experimental Forest Farm in Qingyuan County, Zhejiang Province, China (119°01’25″ E, 27°38’48″ N), with an altitude ranging from 480 m to 510 m. The general situation of the research area is shown in **Figure 1**. The experimental site has a gentle slope. The region has an average annual temperature of 17.6 °C, an annual precipitation of 1721.3 mm, and a frost-free period of 245 days, characteristic of a typical subtropical monsoon climate. The experimental site comprises acidic red soil characterized by a pH of 4.67. Key soil properties include 7.8 g/kg organic matter, 0.45 g/kg total nitrogen, 0.27 g/kg total phosphorus, and available nutrient concentrations of 17.2 mg/kg nitrogen, 37.3 mg/kg potassium, and 1.02 mg/kg phosphorus.

### 2.2 Materials and design

**Figure 2:**
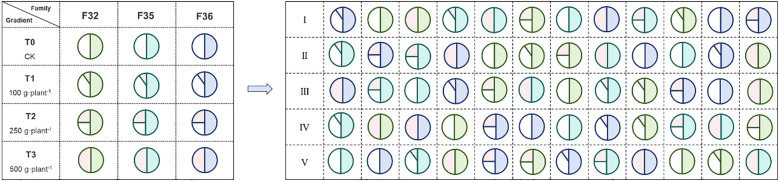
Schematic arrangement of the experimental design. Each compartment contains a total of 40 individual plants, which are planted in 4 rows with 10 plants in each row. F32, ♀6627×♂1003; F35, ♀5163×♂1003; F36: ♀5163×♂6627. T0 (CK) was the unfertilized control; T1, T2 and T3 gradients were applied with 100, 250 and 500 g·plant^-^¹ of diammonium phosphate.

A completely randomized block design with two factors (variety and fertilization) was used. Three *P. massoniana* hybrid varieties were selected: F32 (♀6627×♂1003), F35 (♀5163×♂1003), and F36 (♀5163×♂6627). These hybrid families were generated through artificial controlled pollination within a second-generation breeding population, with all three varieties sharing either a common paternal or maternal parent. The selected genotypes represented distinct geographic provenances: (1) genotype 6627, derived from Jiangxi provenance (central production region), exhibiting 2–3 annual shoot flushes; (2) genotype 1003, originating from Guangdong provenance (southernmost production region), characterized by 3–5 annual shoot flushes; and (3) genotype 5163, representing Zhejiang provenance (northern production region), typically showing 1–2 annual shoot flushes. Four phosphorus fertilizer treatments were established: 0 g·plant^-^¹ (T0, as control), 100 g·plant^-^¹ (T1), 250 g·plant^-^¹ (T2), and 500 g·plant^-^¹ (T3). The applied phosphorus fertilizer was diammonium phosphate ((NH_4_)_2_HPO_4_: containing 46% phosphorus and 18% nitrogen). In March 2020, a measurement forest was established according to the scheme shown in **Figure 2**. The 12 treatment combinations were replicated five times (blocks), with each block containing all treatments randomly arranged. Each treatment plot comprised 40 trees (4 rows × 10 plants, 2 m × 2 m spacing), totaling 2,400 trees (12 treatments × 5 blocks × 40 trees). Fertilization was applied in May 2020–2022 via a continuous circular ditch (15 cm from trunk, 20 cm deep, 10 cm wide) around each tree. Growth traits (fixed growth: predetermined bud flushes; free growth: post-flush shoot elongation) were assessed for all 40 trees per plot (no subsampling). Protective borders of open-pollinated seedlings surrounded each block. Annual weeding ensured minimal competition, with no additional nutrient/water inputs.

### 2.3 Survey and measurement

After the third growing season, tree height, diameter at breast height (DBH), and annual shoot characteristics were measured for each tree. The measurement of annual shoot characteristics was based on the whorled growth pattern of *P. massoniana*. After overwintering, the previous year’s winter buds began to sprout in the following spring. Shoot growth was determined by measuring the length between two spring branch whorls and the number of branch whorls, thereby calculating the annual height growth and shoot number. The growth of the initial shoot (fixed growth) was the distance from the base of the spring branch whorl to the base of the first shoot. For free whorl branches, the measurement was carried out upward in the same way to obtain the annual shoot number and growth. In addition, four individual trees were randomly selected from each treatment combination in each replicate, and the current year’s needles from the upper part of the tree crown were collected and brought back to the laboratory. The needles were deactivated at 105 °C for 30 minutes, then dried at 80°C to constant weight, and their mass was measured for the determination of nitrogen and phosphorus content. Phosphorus content was determined by the molybdenum-antimony colorimetric method, and nitrogen content was determined by the Kjeldahl method (Bremner, 1960; Zhang et al., 2025).

### 2.4 Data analysis

We conducted comprehensive analyses of growth traits, shoot morphology, and needle nutrient content in *P. massoniana* saplings across hybrid varieties and phosphorus (P) fertilization treatments. The descriptive statistics (mean, range, standard deviation) and coefficients of variation were calculated for all measured parameters. The calculation formulas are as follows:


(1) $$\text{Average value}:\bar{x}=\sum xi/n$$

(2) $$\text{Genetic Coefficient of Variation}:\text{GCV}=100\%\times \sqrt{\delta_{G}}/\bar{\;x}$$

(3) $$\begin{array}{c} \text{Environmental Coefficient of Variation}:\text{ECV}\\ =100\%\times \sqrt{\delta_{E}}/\bar{\;x} \end{array}$$

Where xi is the measured value for each individual plant, $\bar{x}$ is the mean value for a trait, *n* is the number of individual plants, $\delta_{G}$ and $\delta_{E}$ represent genetic and environmental variance components respectively, estimated using restricted maximum likelihood (REML) methods in a mixed-effects model framework.

The Shapiro-Wilk test was employed to assess the normal distribution of measured data across different hybrid varieties and phosphorus fertilizer application rates. One-way analysis of variance (ANOVA) and Tukey’s multiple comparison test were utilized to analyze the effects of varying phosphorus fertilizer intensities and varieties on needle nitrogen and phosphorus content. Partial least squares (PLS) regression was employed to examine multivariate relationships between functional traits and nutrient content across P gradients, addressing potential collinearity among predictors. All analyses were implemented in R 4.3.2. Effect sizes (η² for ANOVA, r for correlations) are reported alongside p-values.

## 3 Results

### 3.1 Changes in growth characteristics

**Figure 3:**
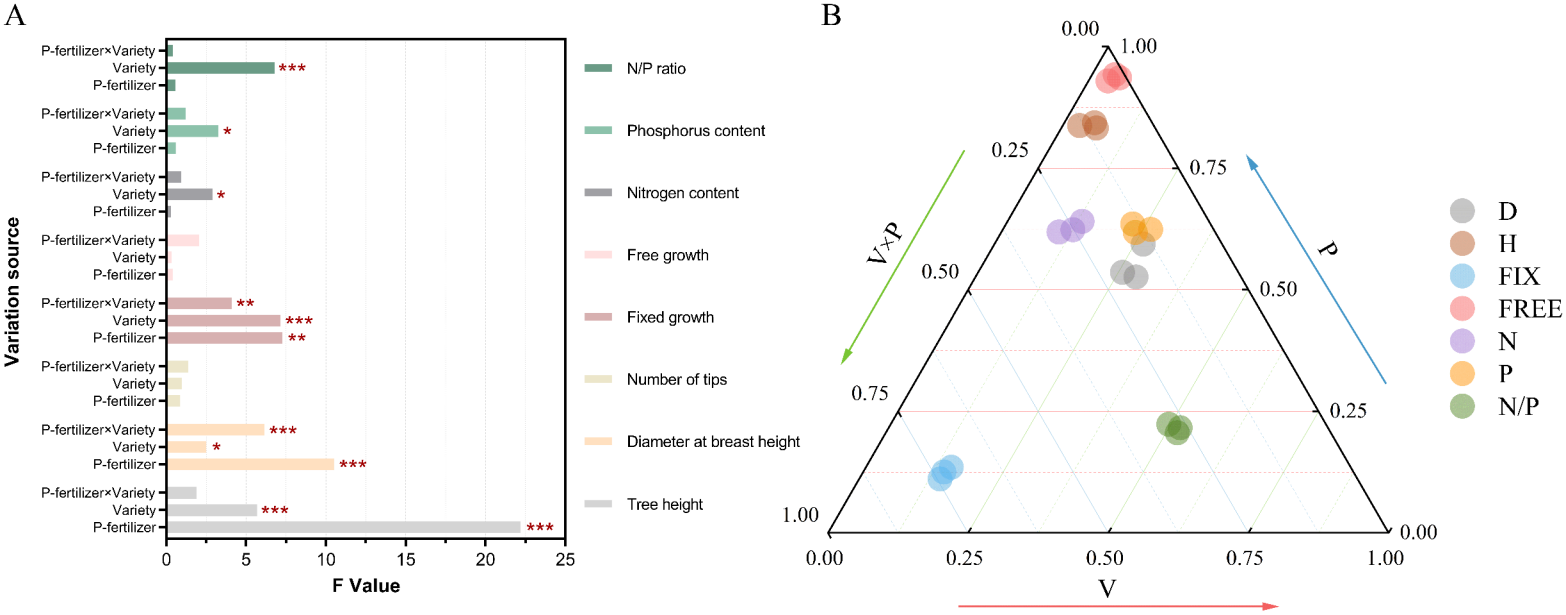
Influence of varieties, fertilization and the interaction effect of the two on the measured traits. **(A)** analysis of variance (ANOVA) for each trait; **(B)** percentage of variance components of genetics, environment, and both interactions for each trait. V, variety; P, phosphate fertilizer; V×P, variety× phosphate fertilizer; D for diameter at breast height, H for tree height, Fix for fixed growth, Free for free growth, N for nitrogen content of needles, P for phosphorus content of needles, N/P for nitrogen/phosphorus ratio of needles.

**Figure 4:**
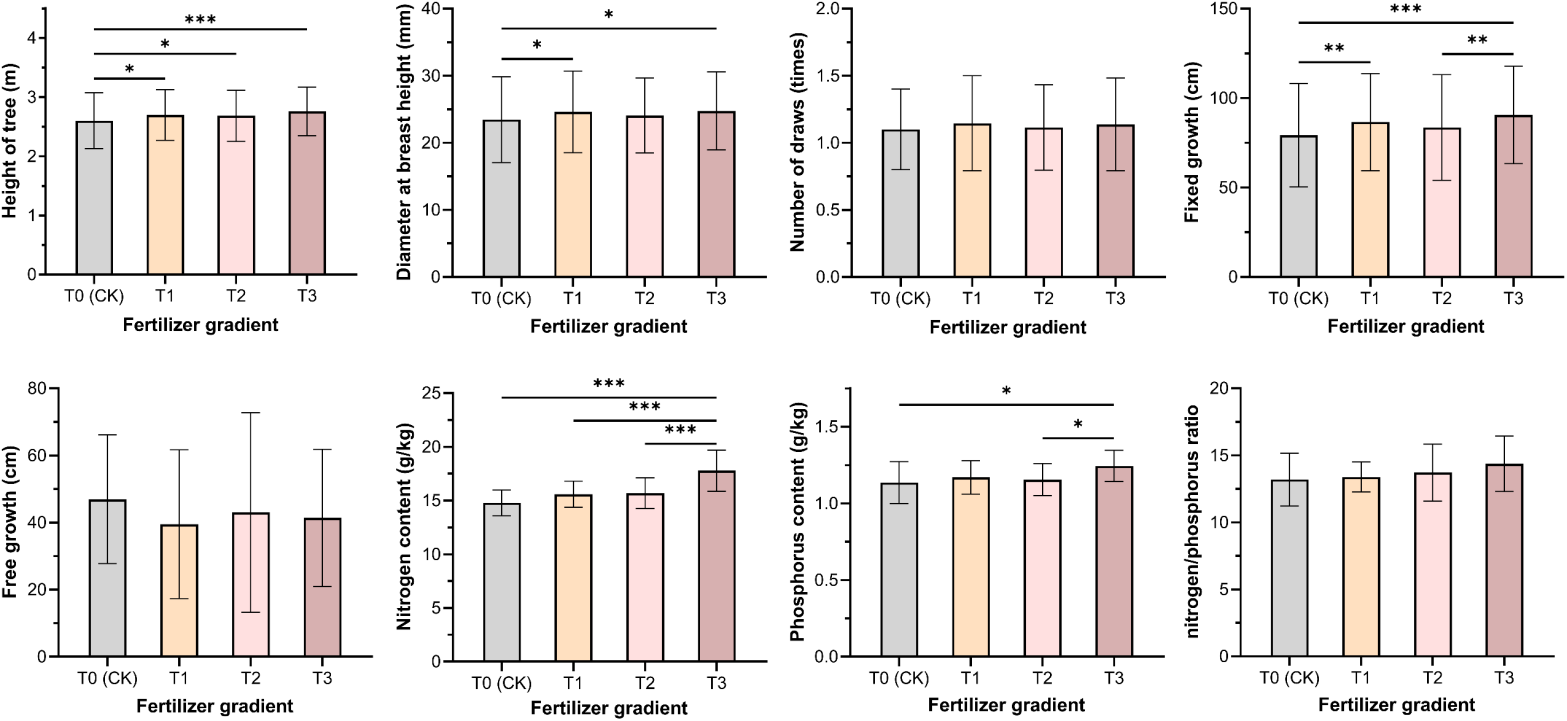
Effects of different phosphorus fertilizer intensities on functional traits and nutrient contents of *P. massoniana*. T0 (CK) was the unfertilized control; T1, T2 and T3 gradients were applied with 100, 250 and 500 g·plant^-^¹ of diammonium phosphate, respectively. * indicates *P*< 0.05; ** indicates *P*< 0.01; *** indicates *P*< 0.001.

After three years of cultivation, the *P. massoniana* exhibited an average tree height of 2.68 m, a diameter at breast height (DBH) of 24.22 mm, 1.12 flushes per year, a fixed growth of 85.02 cm, and a free growth of 42.39 cm. The free growth trait showed the highest genetic coefficient of variation (GCV=11.54%) and environmental coefficient of variation (ECV=52.99%), indicating that it has substantial plasticity. The growth and flushing characteristics of the hybrid varieties responded differently to phosphorus fertilizer application. Significant differences were observed among varieties and phosphorus treatments in tree height, DBH, and fixed growth (**Table 1**, **Figure 3A**). The T3 treatment showed the best performance in tree height, DBH, and fixed growth, with increases of 6.14%, 5.49%, and 14.35%, respectively, compared to the T0 treatment (**Figure 4**). This indicates that higher phosphorus fertilizer content significantly promoted the development of primary growth morphology in *P. massoniana*. No significant differences were found in free growth and annual flushing frequency among varieties and phosphorus treatments, suggesting that the multiple flushing behavior of hybrid varieties was not highly sensitive to phosphorus fertilizer.

**Table 1:** Trait performance and analysis of variance.

Trait	Mean	Variance component			Heritability	Coefficient of variation	
		σ_F_^2^	σ^2Ft^	σ^2E^	*h_f_^2^*	GCV(%)	ECV(%)
Height/m	2.68	0.012	0.020	0.182	0.37	4.09	15.92
Diameter/mm	24.22	0.468	2.232	34.048	0.12	2.82	24.09
Number of tips/times	1.12	0.001	0.001	0.109	0.10	2.82	29.48
Fixed growth/cm	85.02	7.749	30.305	769.965	0.10	3.27	32.64
Free growth/cm	42.39	23.918	60.139	504.497	0.30	11.54	52.99
N g·kg^-1^	15.96	0.093	0.060	2.998	0.26	1.91	10.85
P g·kg^-1^	1.12	0.002	0.002	0.054	0.29	3.80	19.75
N/P	19.06	0.020	0.392	2.692	0.06	1.03	12.00

### 3.2 Changes in conifer nutrient content

The genetic coefficients of variation for needle nitrogen and phosphorus content and the nitrogen-to-phosphorus ratio were relatively small (1.03%-3.80%), lower than the environmental coefficients of variation (10.85%-19.75%) (**Table 1**, **Figure 3A**). No significant differences were observed in needle nutrient content among varieties, but significant differences were found among fertilizer treatments (*P*<0.05). With increasing fertilizer intensity, needle nitrogen and phosphorus content and the nitrogen-to-phosphorus ratio showed an increasing trend (**Figure 4**), indicates that increased external phosphorus supply promoted uptake of both nitrogen and phosphorus in *P. massoniana* needle.

### 3.3 Factor contribution analysis

The distribution of all indicators varied across three dimensions: variety (V), fertilization (P), and their interaction (V×P) (**Figure 3B**). The effect on DBH was in the order of variety effect > interaction effect (V×P) > fertilization (P) effect, with the variety effect contributing 51.22%. The effects on tree height, fixed growth, needle nitrogen content, phosphorus content, and nitrogen-to-phosphorus ratio were in the order of fertilization (P) effect > interaction effect (V×P) > variety effect. The contribution of the fertilization (P) effect ranged from 55.43% to 93.62%, and the contribution of the interaction effect (V×P) ranged from 3.13% to 22.42%. This indicates that fertilization is the dominant factor influencing initial growth and nutrient accumulation, while also being affected by the interaction effect. The interaction effect between variety and fertilization (V×P) had the highest contribution to free growth, reaching 74.1%, suggesting that the flushing behavior of different varieties exhibits heterogeneity in response to fertilization intensity.

**Figure 5:**
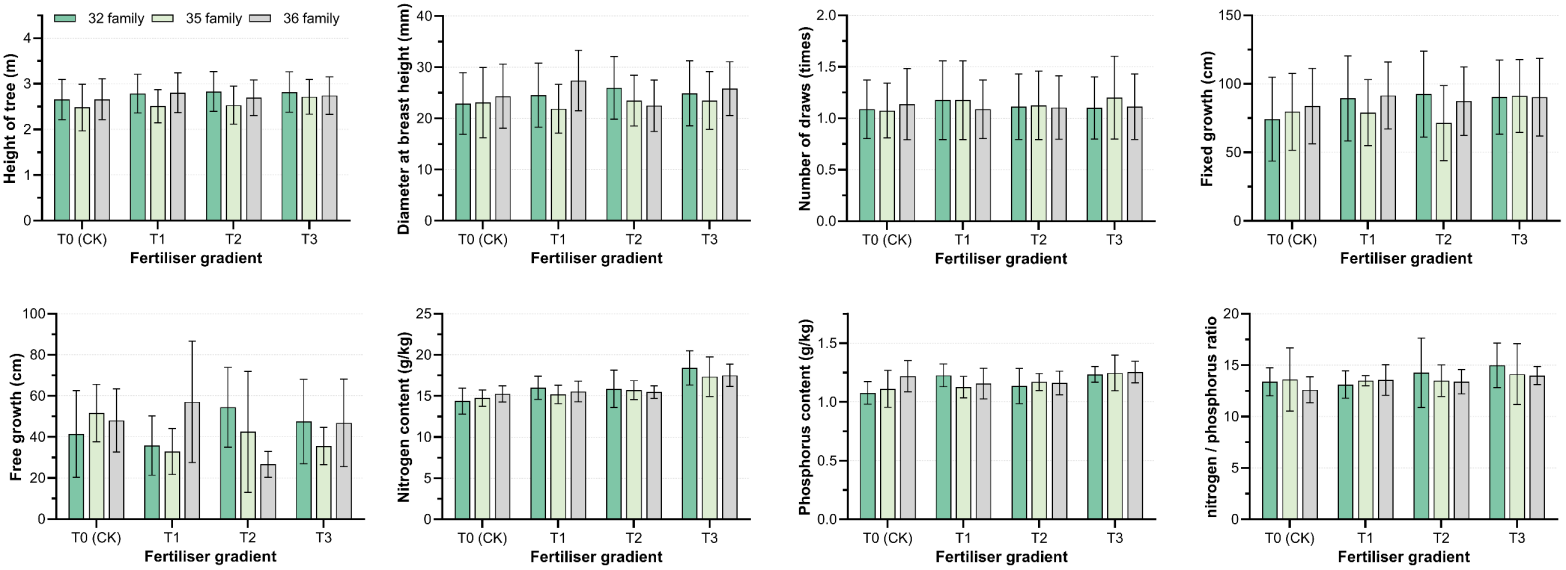
Effects of different phosphate fertilizer intensities and family types on functional traits and nutrient contents of *P. massoniana*. T0 (CK) was the unfertilized control group; T1, T2, and T3 gradients were applied 100, 250, and 500 g·plant^-^¹ of diammonium phosphate, respectively. Only intergroup differences, i.e., differences among family types, are compared in the figure.

The fixed growth of hybrid varieties 32 and 35 increased under fertilization treatments, peaking at T1 and T3 treatments, respectively; in contrast, clone 36 showed the opposite trend. The effects of different treatments on the free growth of each variety varied considerably (**Figure 5**). This indicates that the increase in annual flushing length is attributed to the accumulation of fixed growth and free growth. Hybrid combinations with the same paternal or maternal parent exhibited different response patterns to phosphorus input. However, regardless of low or high phosphorus fertilizer input, the increase in annual flushing length of high-generation hybrid varieties was mainly attributed to the increase in fixed growth.

### 3.4 Correlation between phosphorus fertilizer application and plant traits

**Figure 6:**
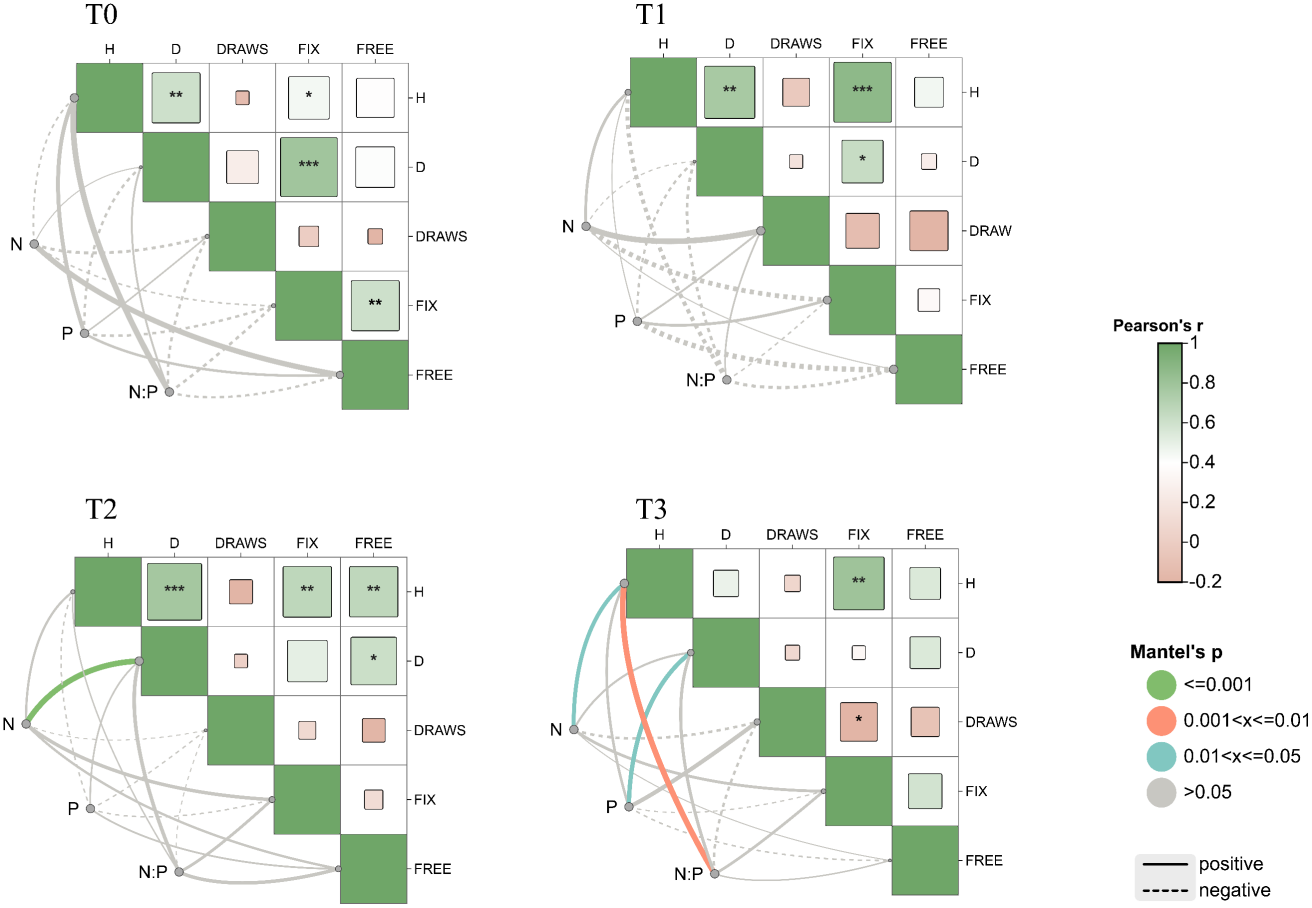
Genetic correlations between functional traits and nutrient content. T0 (CK) was the unfertilized control; T1, T2, and T3 gradients were applied with 100, 250, and 500 g·plant^-^¹ of diammonium phosphate, respectively. * indicates *P*< 0.05; ** indicates *P*< 0.01; *** indicates *P*< 0.001. H for tree height, D for diameter at breast height, Draws for number of draws, Fix for fixed growth, Free for free growth, N for nitrogen content of needles, P for phosphorus content of needles, N/P for nitrogen/phosphorus ratio of needles.

As the amount of phosphorus fertilizer application increased, more correlations were observed between tree height, DBH, and nutrient factors (**Figure 6**). Flushing frequency showed mostly significant negative correlations with other traits (*P*< 0.05), especially under T1 and T2 treatments. The N:P ratio was positively correlated with tree height and DBH, but negatively correlated with other traits. Furthermore, under the T4 treatment, with increasing fertilization, nitrogen, phosphorus, and the N:P ratio showed positive correlations with multiple growth indicators, particularly with tree height (*P*< 0.01). This indicates that the nutritional environment influences the genetic expression of traits, thereby affecting the interrelationships between traits.

## 4 Discussion

### 4.1 Regulation mechanism of phosphorus fertilizer intensity on needle nutrient uptake and utilization

Phosphorus fertilizer application significantly promoted the growth of *P. massoniana* young forests, notably improving traits such as tree height, DBH, and fixed growth (**Figure 3**). As a key nutrient, phosphorus input also increased nitrogen and phosphorus contents in needles, especially phosphorus uptake (**Figure 4**), directly enhancing biomass accumulation and morphogenesis during early growth (Na et al., 2014; Yi et al., 2023). Additionally, phosphorus input indirectly improved nitrogen use efficiency by enhancing soil nutrients or root activity (Mulligan and Sands, 1988). Growth of *P. massoniana* is limited by nitrogen and phosphorus deficiency in southern acidic soils, particularly phosphorus (Xu et al., 2018). This study confirms that exogenous phosphorus alleviates this limitation, improves soil nutrients and plant uptake, and significantly boosts *P. massoniana* growth, especially under low soil phosphorus conditions (Xu et al., 2018). Research has shown that fertilization’s effect on needle nutrients is mainly driven by environmental factors, with fertilization treatments accounting for 52.99%–64.08% of the variation in nitrogen and phosphorus content (**Table 1**), while genotypic influence is relatively limited. This supports Guo W. et al. (2023), who suggested needle nutrient content reflects environmental adaptation more than genotype. Under significant environmental changes, plants exhibit high plasticity in nutrient uptake and allocation, highlighting the importance of fertilization management in forestry (Rubio and Lavado, 1999; Andersen et al., 2017). Fertilization supports optimal growth in variable environments (Freiberger et al., 2013; Niu et al., 2020; Rennenberg et al., 2009).

Under low phosphorus conditions, a negative correlation was observed between DBH and needle phosphorus content (**Figure 5**); as phosphorus supply increased, this shifted to a significant positive correlation, reflecting *P. massoniana* nutrient allocation strategy under phosphorus stress. When phosphorus is limited, the species may prioritize allocation to growth organs (e.g., roots, stems), reducing leaf phosphorus to sustain growth (Turner, 2008). This adaptive mechanism allows plants to maintain growth under stress (DeLoose et al., 2024). By analyzing needle nitrogen and phosphorus content under different phosphorus levels and their correlations with traits like flushing growth and frequency, the study found that nutrient environment strongly influences trait expression and interactions. Without phosphorus fertilizer, traits such as tree height and DBH showed strong positive correlations with other growth traits, which weakened with increased phosphorus input. Thus, appropriate phosphorus fertilization not only alters needle nutrient levels but also indirectly affects flushing traits via the nutritional environment, deepening our understanding of *P. massoniana* growth mechanisms under phosphorus variation.

### 4.2 Effects of genotype-environment interaction on growth traits of *P. massoniana*

This study revealed significant differences in the growth responses of *P. massoniana* hybrid varieties to phosphorus fertilizer intensity, highlighting the critical role of genotype–environment interaction (V×P) in shaping young forest traits. Hybrid combination 32 performed best under moderate phosphorus treatment (T2), with significantly greater tree height and DBH, while combinations 35 and 36 showed better growth under high (T3) and low (T1) phosphorus levels, respectively (**Figure 4**). This demonstrates genotypic variation in phosphorus response and the influence of fertilization intensity on growth potential (Debell et al., 1986). Combination 32, derived from southernmost and central provenances, exhibited high growth potential, while combinations 35 and 36, with northern provenance genes, showed different responses—indicating that growth performance is linked to parent origin. These findings align with Stovall et al. (2011), who reported that genotype × fertilization interaction significantly influenced height and stem growth in slash pine. Other studies confirm that environmental factors, especially fertilization intensity, not only directly affect growth but also interact with genotype responses, emphasizing the importance of G×E effects in forest development (Keller et al., 2018; Zhao et al., 2016).

Tree height showed higher heritability (h² = 0.37) than free growth (h² = 0.30) (**Table 1**), suggesting stronger genetic control over apical growth, while free growth was more influenced by environmental factors, particularly fertilization intensity. Its high plasticity (CV = 54.08%) reflects sensitivity to phosphorus availability, with trees under low phosphorus prioritizing structural growth over flushing—evidenced by the negative DBH–needle P correlation. This resource allocation is hormonally regulated via gibberellin–auxin interactions and elevated ABA under phosphorus stress (Liu et al., 2024). These patterns align with the “source–sink redistribution” model (Suriyagoda et al., 2023), highlighting the need to consider fertilization effects on plastic traits like free growth when managing *P. massoniana*. Genetic variation in flushing traits (h² = 0.30) suggests differential phosphorus sensitivity among clones, shaped by provenance adaptation. Free growth was most affected by variety × fertilization interaction (74.1%), while annual flushing frequency showed low responsiveness, indicating it is less sensitive to phosphorus input. Overall, fertilization interacts with genotype to regulate growth plasticity in *P. massoniana*.

This study found that hybrid combination 32 performed best under moderate phosphorus levels, while combinations 35 and 36 showed better growth under high and low phosphorus, respectively. This suggests that phosphorus concentration has genotype-specific effects on *P. massoniana* growth, with each hybrid maximizing its potential under optimal conditions (Conroy et al., 1990). Such differences likely arise from genotypic variation in nutrient absorption, utilization, and allocation (Liu et al., 2023; McKeand et al., 2023). Therefore, selecting genotypes suited to specific phosphorus levels can enhance fertilization efficiency and forest productivity (Tie et al., 2024; Yang et al., 2025).

### 4.3 Practical significance of optimizing *P. massoniana* plantation management

Shoot-sprouting behavior can reflect the growth rate of *P. massoniana*, with fixed growth and free growth being closely related to genetic and environmental factors, respectively. For hybrid varieties with high-generation genetic improvement, the variation in flushing frequency may not be obvious, but mainly manifested as changes in fixed growth. This means that during the genetic improvement process, the genetic stability of varieties is enhanced, and the buffering capacity against environmental changes is improved, making the flushing frequency relatively stable. Free growth, on the other hand, is more influenced by environmental stimuli. Under different phosphorus fertilizer intensities, the variation in free growth among different clones varied greatly and showed no obvious pattern, reflecting the complex role of environmental factors on it. In forestry practice, this study provides a theoretical basis for “precision genotype-environment matching”. For example, hybrid combination 32 is preferentially planted on sites with moderate phosphorus availability to maximize its root efficiency and phosphorus absorption potential; while hybrid combination 35 is promoted in high-phosphorus soils to avoid phosphorus toxicity risks and optimize resource utilization. This is consistent with the “suitable genotype for suitable site” strategy proposed by Chen et al. (2016), which emphasizes improving plantation productivity and sustainability through precise matching of genotype and environment.

In addition, in actual forestry production, rational fertilization is one of the effective ways to improve the nutrient status and promote the growth of *P. massoniana* (Mao et al., 2021; Guo Q. et al., 2023). More attention should be paid to the regulation of DBH growth, and the timber yield of *P. massoniana* should be increased by optimizing fertilization and selecting suitable genotypes (Qiao et al., 2022). In the future, genomic selection technology should be combined to screen families with high phosphorus response potential, which can accelerate the genetic improvement process of *P. massoniana*. Future research can combine genomics approaches (such as transcriptomics and metabolomics) to analyze the molecular mechanisms of nutrient response in *P. massoniana*, and monitor the feedback effects of fertilization on soil fertility in the long term, to achieve sustainable management of plantations.

## 5 Conclusion

This study revealed the synergistic effects of hybrid varieties and phosphorus fertilizer intensity on the growth and nutrient dynamics of *Pinus massoniana*. Phosphorus fertilizer promoted young forest growth and needle nutrient absorption. With the increase in fertilization intensity, needle nitrogen and phosphorus content and the nitrogen-to-phosphorus ratio showed an increasing trend. Phosphorus fertilization is the dominant factor significantly affecting the initial growth and nutrient accumulation of *Pinus massoniana* hybrid varieties, while the multiple shoot-sprouting behavior of hybrid varieties is mainly affected by the interaction effect of variety and phosphorus fertilizer, showing different plastic responses to phosphorus fertilizer. The contribution rate of the interaction effect between variety and fertilization to free growth reached 74.1%. This suggests that when promoting and applying *Pinus massoniana* varieties with generational improvement, the consideration of the genetic behavior of shoot-sprouting behavior is gradually reduced, and the interaction between varieties and nutrient environment should be given more attention.

## Data Availability

The raw data supporting the conclusions of this article will be made available by the authors, without undue reservation.
